# Progress in Fluorescence Biosensing and Food Safety towards Point-of-Detection (PoD) System

**DOI:** 10.3390/bios13020249

**Published:** 2023-02-09

**Authors:** Saloni Kakkar, Payal Gupta, Navin Kumar, Krishna Kant

**Affiliations:** 1 Institute of Microbial Technology (IMTECH), Council of Scientific and Industrial Research (CSIR), Chandigarh 160036, India; 2 Department of Biotechnology, Graphic Era (Deemed to be University), Dehradun 248002, India; 3 Biomedical Research Center (CINBIO), University of Vigo, 36310 Vigo, Spain

**Keywords:** food pathogen, microfluidic, biosensing, fluorescence microscopy, PoC device

## Abstract

The detection of pathogens in food substances is of crucial concern for public health and for the safety of the natural environment. Nanomaterials, with their high sensitivity and selectivity have an edge over conventional organic dyes in fluorescent-based detection methods. Advances in microfluidic technology in biosensors have taken place to meet the user criteria of sensitive, inexpensive, user-friendly, and quick detection. In this review, we have summarized the use of fluorescence-based nanomaterials and the latest research approaches towards integrated biosensors, including microsystems containing fluorescence-based detection, various model systems with nano materials, DNA probes, and antibodies. Paper-based lateral-flow test strips and microchips as well as the most-used trapping components are also reviewed, and the possibility of their performance in portable devices evaluated. We also present a current market-available portable system which was developed for food screening and highlight the future direction for the development of fluorescence-based systems for on-site detection and stratification of common foodborne pathogens.

## 1. Introduction

Food safety is an assurance for access to healthy and safe food for sustaining life and good health. To ensure food safety, food hygiene must be undertaken in order to preserve the nutritional value of food and protect it from microbial attack from production to consumption. This food safety must ensure the nutritional requirement of the public and, at the same time, it must not expose them to any foodborne illness. Currently, malnutrition and foodborne disease are the major food-related concerns in global population. To avoid foodborne disease, timely detection of pathogens in the food responsible for toxin production and disease is necessary. Food may contain microbes in the form of bacteria, fungus, protozoa, or viruses that are responsible for causing hundreds of diseases from mild through to severe. The United States reported an outbreak of foodborne infections, particularly bacterial infections associated with fresh farm produce in multiple states from 2010 to 2017 [1]. Likewise, a retrospective study was performed to mark the status of foodborne diseases (involving enteric bacteria) in South Africa, from 2013 to 2017 where the presence of *Salmonella* species, *Escherichia coli*, *Bacillus cereus, Listeria monocytogenes*, and *Clostridium perfringens* were reported in food samples [2].

Foodborne diseases are consequences of harmful toxins or other chemical substances produced by naturally occurring microbes in the food material which, upon entering the host body, lead to digestive-system dysfunction [3]. The escalation in foodborne diseases and associated mortality is a result of the prevalence of harmful pathogens in food due to the evolution in agricultural practices, food production and storage methods, under-cooked animal products, ready-to-eat mixes, and, most importantly, globalization of the food trade [4]. In pursuance of safe food supply and reduced incidence of foodborne diseases, an early, quick, and accurate detection of pathogens in food items is required [5]. A number of conventional methods for detection of foodborne pathogens are available. These are based on culturing microbes on differential media, biochemical characterization, sequencing, and characterization through HPLC, MS, PCR, etc. [6]. However, these methods are expensive, time-consuming, and unwieldy, thus restricting their use in point-of-care (PoC) applications [7]. In the food industry, rapid detection of microbes, even at very low numbers in food samples (both raw and processed), is of utmost importance in order to ensure the food quality and safety [8,9]. With the advancement in point-of-care detection methods, researchers have been now able to offer ASSURED (affordable, sensitive, specific, user-friendly, rapid and robust, equipment-free, and deliverable) technologies to the users [10]. The signals in PoC applications are usually fluorescence-, colorimetric-, or electrochemical-based and are simple and easy to interpret/read [11]. Nevertheless, PoC applications have evolved greatly, with advancements still continuing to address challenges in translation of methods from laboratory- into industrial-application detection systems. Some of the key challenges which need attention are sensitivity, multiplexing, quantification, and multi-functionality.

**Figure 1:**
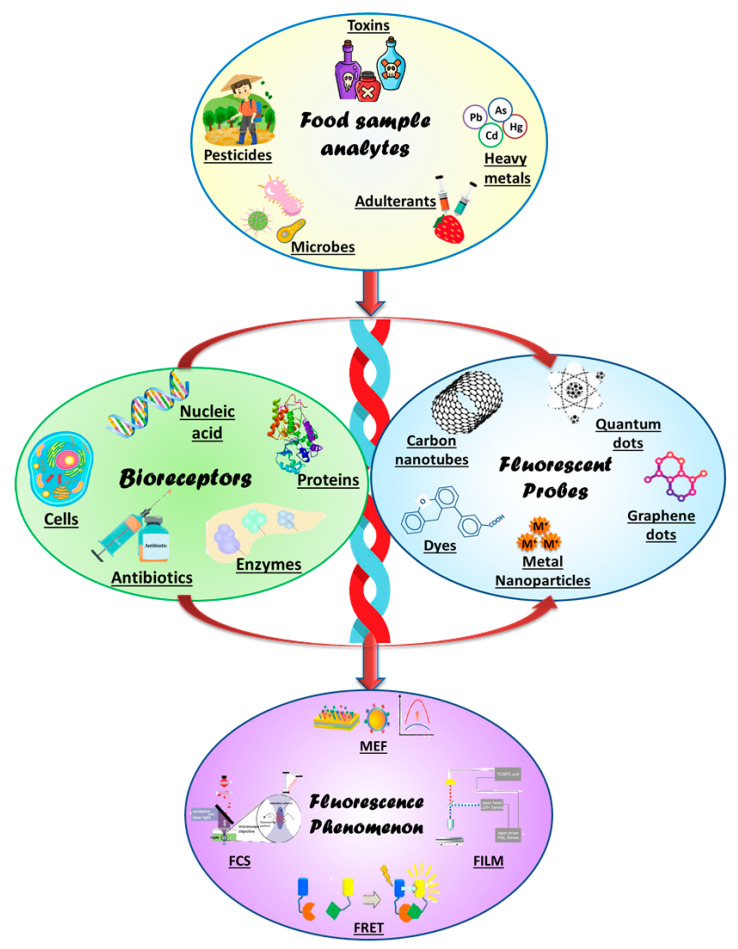
The schematic diagram presents the fundamental components for designing a fluorescence biosensing platform for food sensing. The food analytes (microbes, pesticides, adulterants, pollutants) are detected by using the specific bioreceptors (proteins, enzymes, cells, DNA) generated against various toxins/pesticides/adulterants, etc. These bioreceptors are coupled with bioprobes (nanoparticles, CNT, graphenes, quantum dots, etc.) that are fluorescently active to generate a fluorescent signal (MEF, FERT) response.

Indeed, rapid and sensitive detection methods have evolved greatly, and are still evolving, making them highly sensitive, compact, and reusable with almost no detection time. In the present review, we have summarized the fluorescence biosensing basics of a variety of fluorescence-sensing methods. The review describes fluorescence biosensing materials stretching from nano to molecular to protein-based biomolecules. Further, the different materials used for integrating fluorescence-biosensing and fabrication-detection systems are also been described. The various sequential aspects and approaches of fluorescent-based biosensors are summarized under the schematic presentation in Figure 1. The figure is a detailed flow-chart for detection of food microbes/toxins/ions with the help of bioreceptors such as DNA/proteins generated against these food analytes conjugated with fluorescent active bioprobes viz. nanoparticles/graphene/quantum dots, etc. The fluorescent signal output thus generated can be in the form of FCS, FRET, or FILM; each of these components is discussed in the following sections of the review.

## 2. Fundamental Aspects of Fluorescence Biosensing

Among the variety of available sensing options, fluorescence biosensors are the most promising due to their high sensitivity and selectivity which extends their usefulness in biosensors for clinical and environmental monitoring. When a substance absorbs light of higher energy/shorter wavelength and emits low-wavelength light which is a very-short lived (10^−9^ to 10^−8^ s), this light is called fluorescence [12]. Fluorescence-based detection in biosensors is beneficial for aspects such as sensitivity, signal detection limits, and accuracy.

Developments in nanotechnology have also revolutionized the field of fluorescence biosensing and improved the specificity and sensitivity of the analyte to nano-levels. An example of this is fluorescence-based detection using a cleavable hairpin beacon coupled with LAMP (loop-mediated isothermal amplification) to probe the presence of the *Borrelia burgdorferi* recA gene where the system showed a sensitivity of detecting nearly 100 copies of the gene in 25 min [13]. This sensitivity is many folds higher than that of traditional organic dyes and other fluorescent probes. Among the variety of nanomaterials, quantum dots and carbon nanotubes/carbon dots have gained special attention due to better compatibility, higher surface-to-volume ratio, better chemical and thermal stability, and faster detection. The carbon-nanoparticle-based fluorescence detection of ferrocyanide ion in food samples such as salted foods (radish, cucumber, cabbage) was achieved with a detection limit of as low as 3 ng/mL [14]. Another efficient and sensitive quantum dot (QD)-based fluorescent system to probe the presence of thiram in food samples was reported. The QD consisted of mesoporous silica loaded with a gold nanocluster with the LoD of 0.19 ng/mL [15]. All these features favor its application in point-of-detection (PoD) devices which have maximum demand in diagnostics where sensitivity, specificity, and user-friendly quick response are needed for analyte detection. To utilize these fluorescent labels in biosensing applications, the fluorescence measuring/sensing/estimating phenomenon also need to be understood, and this is elaborated in the following section.

## 3. Fluorescence Biosensing Materials

**Figure 2:**
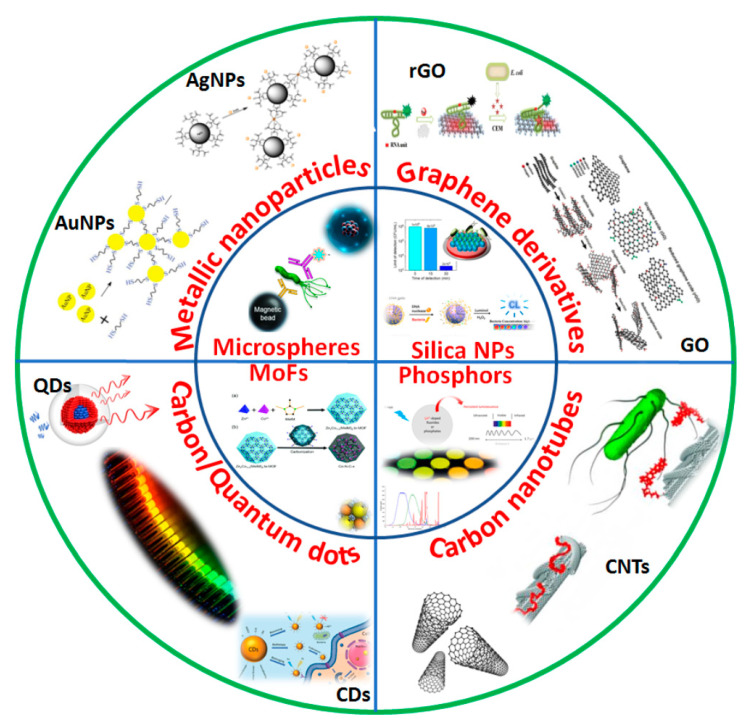
Variety of nanomaterials and their surface modifications used in biosensing of food toxins/pathogens like metallic nanoparticles such as AgNPs (Silver nanoparticles), AuNPs (Gold nanoparticles), carbon nanomaterials viz. QDs (Quantum Dots), GO/rGO (Graphene oxide), carbon nanotubes and other MoFs (Metal organic frameworks), Silica nanoparticles, Microspheres, Phosphors, etc. materials.

With the advancements in the field of nanobiotechnology, fluorescence-based detection methodologies have replaced conventional organic dyes with nanomaterials as detection labels due to their superior optical properties viz. a wide range of excitation and emission wavelengths and brighter fluorescence with better photostability [16]. Figure 2 summarizes a wide range of nanomaterials that are used for fluorescent based point-of-care biosensing of food analytes such as varied nanoparticles, graphene derivatives, metal organic frameworks, carbon-based nanomaterials, etc. Moreover, traditional fluorescence biosensors employing organic dyes do not offer low detection limits, hence compromising the sensitivity of the assay due to limited quantum yields and low receptor binding ratio of dyes. The potential biocompatibility of fluorescent nanomaterials owing to their physico-chemical properties enhances the performance of biosensors, delivering low-cost and portable point-of-care fluorescence sensing of food contamination. Additionally, these fluorescent nanomaterials will impart a solid support system for biosensing conjugated with multiple probes with high labeling ratio yielding high sensitivity [17]. Nanomaterials as fluorescent packets are advantageous in having tunable optical properties with greater quantum yield. Hence, considering the applications of fluorescent nanomaterials in food sensing, we will discuss the major advances and improvements of various nanomaterials that are currently being used for designing fluorescence biosensors. The applications of different nanomaterials and the enhancement of their limits of detection in the system are summarized in Table 1. A list of recent studies of metal nanomaterials and carbon-based nanomaterials along with some other nanomaterials is featured in the table with comparison between their limits of detection for analyzing a wide range of food analytes.

### 3.1. Nanomaterials

**Metallic nanoparticles** acquire quantum mechanical effects such as photoluminescence and the photobleaching resistance of gold nanoparticles encourages the development of in vivo fluorescence biosensors with less toxicity [17]. Gold nanoparticles are excellent FRET-quenchers due to their surface plasmon in visible range, which causes strong absorption and scattering with huge extinction coefficients [18]. A study has reported the gold-nanoparticle-based combined fluorometric and spectrophotometric biosensing of biogenic amines in poultry meat samples. The excitation and emission of histamine conjugated with gold nanoparticle was measured and showed 50 times enhanced fluorescence compared to histamine alone [19]. Silver nanoparticles are great substrates for metal-enhanced fluorescence (MEF) as they contribute towards enhanced fluorescence signal intensity lowering the detection limit of bioassays. These particles are also known to be great acceptors in FRET, and they even promote the efficacy of the assays as FRET pair enhancers. A recent work published by Kato et al. demonstrated a one-pot method for stable coating of silver nanoparticles with a thiolated polymer to form polymeric shells that behaved as an excellent quencher. Great potential for increase in fluorescent plasmonics was observed along with efficient masking of fluorescence quenching with polymer-coated silver nanoparticles [20].

**Carbon nanotubes (CNTs),** which have a unique arrangement of sp2 hybridized carbon atoms that form a π-conjugated network, have been explored in depth in developing fluorescent biosensing assays. The ability of CNTs to quench the fluorescence of organic dyes or quantum dots in the NIR region is combined with photoluminescence properties through dynamic energy transfer [21]. Chen et al., reported on the development of an acetylcholine-based, cost-effective, and sensitive electrochemical sensor to detect pesticides in food samples. The assay used MWCNTs that increased surface area for effective electrochemical polymerization, yet maintained the enzymatic activity, exhibiting a stable response towards multiple real samples such as carbonated drinks, milk, orange juice, and beer [22].

**Quantum dots (QDs)**, also known as semiconductor crystalline materials, are novel fluorescence materials with quantum confinement effect, good photostability, and effective biocompatibility, and they possess composition-based emission tunability [23]. With large Stokes shift and flexible fluorescence, their applications include biosensing, biomedicine, and optoelectronics [24]. QDs possess superior attributes of broader excitation with narrow emission spectra, longer time of fluorescence, and 100 times higher molar extinction coefficient than conventional organic dyes [25]. All these exceptional properties have led to the development of highly efficient and stable optical biosensing systems enabled via QD-based FRET systems. QDs directly enable the sensing phenomenon by enhancing or quenching via direct adsorption/chelation/interaction of specific conjugated bioreceptors or metal ions [26]. Many studies have reported the applicability of QDs and their conjugated derivatives in developing fluorescence-based platforms for pathogen sensing and food safety [27,28,29].

**Graphene**-based nanomaterials are graphene sheet, graphene oxide (GO), and a reduced form of graphene-oxide nanosheet (rGO). Graphene and its derivatives possess outstanding ability in quenching fluorescent dyes so they are used as potential energy acceptors in designing fluorescent sensors. They are often combined and conjugated with fluorophores such as QDs and UCNPs in the form of FRET pairs [30]. Various aspects of biomedical applications such as chemi-sensors, electrochemical sensors, and fluorescent biosensors serving either as quenchers or fluorophores have been explored [31]. A study reported a conjugated system of QD–aptamer–GO for detecting β-lactoglobulin in food samples [32]. Other nanomaterials, such as metal organic frameworks, up-conversion nanoparticles, silica nanoparticles, and phosphors, also contribute to the development of point-of-care fluorescent biosensing technologies for food safety. Various food analytes and the detection limit for these analytes are summarized in the Table 1. In conclusion, all these nanomaterials, with their advanced properties have resulted in the development of efficient fluorescent biosensors for food safety. Table 1 provides a comparative analysis of the bioreceptors employed for detection and their LoD. Although major nanomaterials exhibiting fluorescent properties have been discussed in this review, high-end nanohybrids incorporating conjugated nanomaterials, magnetic nanoparticles, and co-embedded manipulations that are easily fabricated have been reported to be upcoming substitutes. Moreover, depending upon the fluorescent phenomenon being used, such as quenching/masking or fluorescence enhancement involved in food sensing, the particular nanomaterial is selected for its respective application providing improved sensitivity compared to traditional dyes.

**Table 1:** List of food-analyte sensing by various types of nanomaterials (2018–2022).

Nanomaterial	Analyte	Biorecognition Element	LoD	Ref.
**Metal Nanoparticles**				
Gold nanoparticles	Salmonella typhimurium	DNA aptamer	36 CFU/mL	[33]
Gold nanoparticles	Dipicolinic acid	Eu^3+^ ion/gold nanocluster	0.8 μM	[34]
Gold nanoparticles	Histamine	Gold nanoparticles	2.04 nM	[35]
Silver nanoparticles	Melamine	Polyethyleneimine–silver nanobioprobe	132 nM	[36]
Silver nanoparticles	Staphylococcal enterotoxin A	DNA aptamer	0.3393 ng/mL	[37]
Silver nanoparticles	Fe^+3^ ions	Vitamin B12-functionalized biological silver nanoparticles (FAgNPs)	2 mg/L	[38]
Copper nanoparticles	Zearalenone	Antibodies	16.0 μg/kg	[39]
Platinum nanoparticles	Hypoxanthine	Platinum nanoparticles	2.88 μM	[40]
Tungsten nanoparticles	Maltose and sucrose	Fenugreek β-amylase functionalized tungsten disulfide nanoparticles	0.052 and 0.096 mM	[41]
Palladium nanoparticles	Tetracyclines	Graphene quantum dots/palladium nanoparticles	45 ng/mL	[42]
**Carbon-based nanomaterials**				
Carbon nanotubes	*Escherichia coli* O157:H7	Carbonyl iron powder/MWCNT-DNA aptamer	3.15 × 10^2^ cfu/mL	[43]
Carbon nanotubes	Patulin mycotoxin	Carboxyfluorescein dye MWCNTs–DNA aptamer	0.13 μg/L	[44]
Carbon nanohorns	Fipronil	FAM–aptamer with oxidized single-walled carbon nanohorns	3 nM	[45]
Carbon dots	Tartrazine	Fluorescent carbon dots	12.4 nM	[46]
Carbon dots	Tetracyclines and Al^3+^	Fluorescent carbon dots	0.057–0.23 μM and 0.091 μM	[47]
Carbon dots	Ascorbic acid	Carbon Dots/Fe^3+^ composite	3.11 μmol·L^-1^ μmol/L	[48]
Quantum dots	Acrylamide	DNA aptamer	2.41 × 10^−8^ M	[49]
Quantum dots	Histamine	Carbon quantum dots with peptide	13 μg/kg	[50]
Quantum dots	Biogenic amines	Carbon dots/yellow fluorescent CdTe quantum dots	1.259-5.428 μM	[51]
Graphene oxide	β-lactoglobulin	DNA aptamer	96.91 μg/L	[32]
Graphene quantum dots	Formaldehyde	Graphene quantum dots	0.0515 μg/mL	[52]
Graphene oxide	Zearalenone and ochratoxin A	Cy3 aptamer and Alexa Fluor 488 aptamer	1.797 ng/mL and 1.484 ng/mL	[53]
**Other nanomaterials**				
Silica nanoparticles	Thiram	Mesoporous silica with gold nanoparticles	0.19 ng/mL	[15]
Silica nanoparticles	Aflatoxin B_1_	DNA aptamer	0.13 ng/mL	[54]
Up-conversion nanoparticles	Staphylococcus aureus	Aptamer-functionalized gold nanoparticles	10.7 CFU/mL	[55]
Up-conversion nanoparticles	Histamine	Up-conversion nanoparticles	7.34 mg/L	[56]
Metal organic frameworks (MOF)	Acrylamide	6-carboxyfluorescein-labeled aptamer (FAM-ssDNA)	1.9 nM	[57]
Metal organic frameworks	Tetracycline antibiotics	Luminescent MOF	0.28–0.30 μM	[58]
Metal organic frameworks	Ethanolamine	Zeolitic imidazolate framework-8/FAM-aptamer	17.86 pM	[59]
Phosphors	Zearalenone in cereals	Black phosphorus–gold nanocomposite	2 μg/kg	[60]

### 3.2. Nucleic-Acid-Based Molecular Markers

Fluorescent-based molecular markers such as DNA/mRNA covalently conjugated with fluorophore are used for sensing applications as they selectively bind to functional groups of target molecules [61]. Fluorescent DNA/RNA can also be generated by use of 2-aminopurine (for adenine) or isoxanthopterin (for guanine) nucleobase analogs and used as efficient molecular recognition elements (MREs) for developing target-detection systems [62]. Generally, fluorescent nucleic acids are classified based on their structures that begin with detecting SNP based on duplex formation. Another structural analysis of homoadenine and A-cluster systems demonstrated their applicability in three-way-junction (3WJ) probes for targeting miRNA. Moreover, G-quadruplexes with their G-rich sequences form fluorescent probes or detection of targets. Most important is the selectivity and specificity of a new group of fluorescent molecular beacon (MB) systems towards target sequence [63]. MBs are highly specific single-stranded DNA fluorescent probes that are dual modified at one end with fluorophore (F) and at the other end with a quencher (Q), leading to their applicability in detection systems [64]. An MB can acquire an open structural state where the quencher is away from the fluorophore, spatially restoring the fluorescence that generally happens in the presence of target and closed state where the fluorophore and quencher come into close proximity, diminishing the fluorescence. A recent study reported the detection of signature molecules of food-borne pathogens using the FRET mechanism of MBs, QDs, and nanoscale quenchers [65]. Moreover, MB-based multiplex real-time PCR studies have been reported for detection of various food pathogens [66,67,68]. Evolving from the inherent attribute of nucleic acid to form Watson–Crick duplex structures to detect complementary nucleic acid strands, there have been ground-breaking discoveries of generating affinity nucleic acids possessing specific binding properties [69]. Over the last decade, single-stranded DNA/RNA aptamers as a versatile class of bioreceptors, have been introduced. Their ease of synthesis and excellent biofunctionalization properties enable efficient fluorescent sensing **[70,71]**. The fluorophore is conjugated with an aptamer as a labeled/non-labeled moiety and target detection is determined by excitation-light interaction with the bioreceptor reflecting fluorescent intensity [72]. A recent study reported a signal-on fluorescent MB-aptamer-based sensor for rapid detection of mercury in food samples [73].

Comprehensively, as compared to the classical conventional bioreceptors for sensing applications, aptamers pave novel avenues for designing fluorescent detection strategies due to their exceptional properties that allow bioconjugation with a large variety of compounds. They offer high sensitivity for detection of target analytes enabling specific biorecognition abilities that promote potential sensing applications.

### 3.3. Antibodies

Fluorescent immunoassays generally use antibodies covalently linked with fluorochrome that absorbs light and emits at another wavelength as detection reagents. In point of fact, a few years ago, the novel concept of a fluorescent immunosensor, a Quenchbody, also known as a Q-body, was introduced by Ueda and colleagues. The key aspect of this technology comprises antibody-labeling of the N-terminal region/antigen-binding fragment (Fab) of an antibody with fluorescent dyes, delivering enhanced fluorescence when antibody–antigen interaction occurs [74,75]. Fluorescent dyes such as TAMARA, ATTO520, and rhodamine are conjugated with variable regions of antibodies via flexible linker peptides [76]. These fluorescent-labeled antibodies are utilized in designing lateral-flow test cards for sensing food contaminants. Alongside, smartphone integration with fluorescent detectors have been successfully used as efficient point-of-care systems for sensing pathogenic bacteria in food samples [7]. Huang et al., in 2017, reported a protein-sensing platform employing a combination of graphene-oxide sheets conjugated with antibodies that displayed quantitative quenching of fluorescent signals [77]. Another fluorescence-antibody-labeled sandwich immunoassay was reported using chitosan–cellulose nanocrystal membrane for rapid detection of *Listeria monocytogenes* in food samples [78]. Over the recent years, the advancements in antibody-based detection techniques have increased due to immunological modifications, resulting in effective food-sensing applications.

### 3.4. Proteins/Enzymes

Several protein-based assays have employed studies of protein modification/interaction, kinase activity, time-bound fluorescent assays, detection of toxins/adulterants, identification of viral antigens/pathogens, etc. These have incorporated fluorescent dyes viz. Cy5, BEBO (cyanide dye), lanthanides (e.g., Eu^3+^, Sm^3+^, Tb^3+^ and Dy^3+^), SYBR green, NanoOrange, and RiboGreen that have been utilized in fluorescent biomolecular assays such as bimolecular fluorescence complementation (BiFC), lanthanide fluorescent immunoassay, fluorescent-dye-based assay, chemifluorescent enzyme-linked immunosorbent assay (ELISA), real-time immuno-PCR, immuno-detection, and sandwich fluoroimmunoassay [79]. Green fluorescent protein (GFP) or yellow fluorescent protein (YFP) have quite often been used as reporter conjugates/markers in the detection of pathogens for food sensing, helping in enumerating/tracking of bacterial cells. For complex sample preparation, fluorescent proteins with their longer wavelengths avoid the limitation associated with fluorescent dyes. Along with GFP and YFP, R-phycoerythrin (PE) isolated from red algae is also used as stable fluorescent protein [80]. A study has reported a novel TurboGFP expression vector for labeling of Yersinia species *Y. enterocolitica* biova*r* 1A, biovar 2, biovar 4, and *Y. pseudotuberculosis.* After being transformed with the vector, these bacteria expressed fluorescence of bright green color that could be seen with the naked eye [81]. Similarly, a fiber-optic toxicity biosensor incorporating GFP label modification of *Escherichia coli* was designed for detection of hazardous heavy metals such as Cu(II), Cd(II), Pb(II), Zn(II), Cr(VI), Co(II), Ni(II), Ag(I), and Fe(III) and their toxicity in the samples [82]. Apart from proteins, certain enzyme-based sensors utilizing peroxidase (HRP), glucose oxidase, lactase, urease, alkaline phosphatase, etc., integrate fluorescent properties of coenzymes that absorb light or substrates for catalytic reactions play a crucial role in sensing of food, toxins, pathogens, etc. Enzymes, being different moieties than generalized bioreceptors, are not directly involved in detection of analytes but they amplify the signal by catalyzing certain reactions. Likewise the fact that they only need a substrates in order to work, but are not affected by the working medium/environment, makes them outstanding as potential substitutes for sensing and food-monitoring applications.

## 4. Integration of Fluorescence Biosensing for Microbe Detection

Over recent decades, development of fluorescence-based detection of pathogenic microbes has accelerated, with the development of direct and rapid point-of-care testing techniques that maintain proper safety assessments. Fluorescence biosensing has the well-established advantages of immediate response time, highly sensitive detection, easy labelling of fluorophore with functional groups, localized fluorescence signals that provides visible output using multicolor dyes, and multiplexed detection assays [83]. For decades culture-based methodology was the gold-standard. It offers low-cost, equipment-free, and easy-operational detection assays. However, its time constraint compromises rapid and on-site detection. Then, PCR (polymerase chain reaction) and LAMP (loop-mediated isothermal amplification) assays were developed, offering high sensitivity and rapid bacterial detection. However, several bottlenecks related to expensive instrumentation, false-positive results, and the need for trained manpower also restricted their applicability for point-of-care microbial detection systems. Moreover, immunological techniques, such as ELISA, that are increasingly recommended for pathogen detection due to their sensitive antigen–antibody interaction, also have shortcomings of cross-reactivity, longer durations for result processing, and complex sample processing [84]. Therefore, to avoid the limitations of the aforementioned methodologies, high-performance novel fluorescence-based biosensing techniques were introduced. These are sensitive up to an ultralow level microbial concentrations and satisfy the high demand for food safety. Here, we will focus upon these fluorescence-based bioassays comprising microarray/biochip assays, microfluidics assays, paper-based hand-held devices, and lateral-flow devices.

### 4.1. Microarrays

Fluorescence-based microarrays comprise a microtiter plate, a glass slide onto which the sample protein is bound in an array, and fluorescently labelled probe molecules which are added to deliver chemiluminescence or a colorimetric signal readout. The fluorescence-labelled probe interacts with the immobilized protein samples releasing a fluorescent signal that is further scanned by laser for detection. The biochemical activity of protein-sensing is generally studied using three types of array—analytical, functional, and reverse-phase protein microarrays—that are consolidated for pathogen-sensing, ensuring food safety. Studies have shown that the bead/suspension array technique provides detection of bacterial/plant toxins, mycotoxins, and pesticides in food using microsphere beads conjugated with biomolecules such as DNA oligonucleotides/proteins labeled with fluorescent dye. The DNA microarray technique comprises immobilization of cDNA probes on a solid matrix onto which PCR-amplified fluorescent-labeled DNA molecules are hybridized. Their interaction generates a signal, allowing detection of known probes on the microarray. DNA-microarray-technology applications have been extended to a great extent for detection of food pathogens. Fluorophores that are generally incorporated for labeling of probes are Cyanine5/Alexa Fluor 647 (excitation at 650 nm/emission at 668 nm), Cyanine3/Alexa Fluor 555 (excitation/emission values at 550/568 nm), and bacterial-species-specific antibody-labeled and biotinylated DNA/RNA aptamers in combination with fluorescence-labeled streptavidin [85]. An in situ generated biochip was designed for detection of food pathogens present on freshly cut vegetables and fruits. Specific sequences of Vibrio *parahemolyticus*, *Escherichia coli* O157:H7, *Salmonella typhimurium*, *Staphylococcus aureus*, and *Listeria monocytogenes* were identified using tilling array probes in a hybridization array. The assay produced strong amplification signals with detection limit of 3log CFU/gm on freshly cut lettuce and cantaloupe in 24 h time detection [86]. Another work studying the amplification of foodborne-pathogen sensing on microarray comprised Cy5-dye-labeled double biotin DNA linkage and detection antibody as Cy5–Ab complex. Simultaneous detection of *Salmonella* and *E. coli* was achieved as visual screening followed by fluorescence-based quantification. A detection limit of 10^3^ CFU/mL and 9 CFU/mL in buffer and real food was achieved via visual screening and quantification of fluorescence intensity [87].

### 4.2. Microfluidic Devices

Microfluidics technology is considered to be a multidisciplinary technique interlinking several aspects of science including biochemistry, fluid dynamics, material science, physics, engineering, nanotechnology, chemistry, microtechnology, and biotechnology. It has been introduced as a novel point-of-care testing device in biosensing, providing large surface-to-volume ratio and making it a portable technology [88]. Fluorescence-based detection on microfluidic chips comprises bioluminescence, laser-induced fluorescence, immunofluorescence technique, and chemiluminescence, and the unique combination of these biochips with fluorescence detectors efficiently promotes sensitive detection of food-borne pathogens [89].

The fabrication of microfluidic-based devices comprises manufacturing technologies using silicon, glass, polymer (polydimethylsiloxane:PDMS) and ceramic that employs a photolithography method integrating mass production by micro electro-mechanical systems (MEMS). Generally, there are three versions of microfluidics: (a) continuous-flow, (b) droplet-based, and (c) digital, and their fabrication employs wet-etching, molding, sanding, laser, and milling techniques. Microfluidic fluorescence sensors need to maintain excitation spectra slightly different to the emissions in order to obtain complex spatial arrangement in glass-based microfluidics, while polymer microsystems often use PDMS that incorporates molding, layer structuration, or 3D printing which is an inexpensive method. Lastly, ceramic was primarily utilized in microelectronics due to its significant features of designing 3D structures in low-temperature cofired ceramic (LTCC) [90]. PDMS were also applied as the surface for capturing bacteria. A recent study presented a 3D PDMS sponge fabrication utilizing salt crystals as the scarifying mold and the inner surface of the PDMS sponge was functionalized by apolipoprotein-H (ApoH), as universal ligand to capture both Gram-positive (*L. monocytogenes*) and Gram-negative (*Salmonella* spp.) bacteria, in combination with a microfluidic bioreactor. The capture proficiency was found greater than 70% for both targeted pathogens with an LoD of 10^3^ and 10^4^ CFU/mL for *Salmonella* spp. and *L. monocytogenes*, respectively [91].

**Figure 3:**
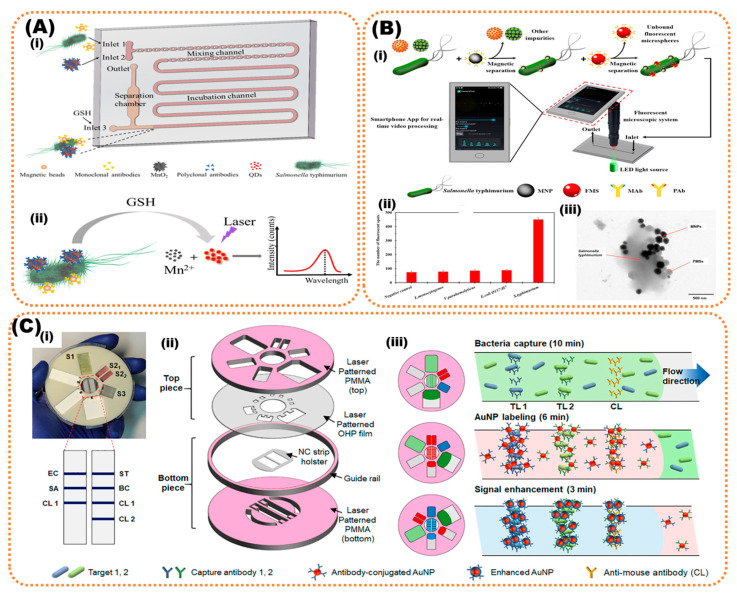
(**A**) PDMS microfluidic-platform-based study reports QD fluorescent-probe-based detection of *Salmonella typhimurium*. (**i**) Schematic presentation of microfluidic channel with inlet and outlet and presentation of the experimental process and (**ii**) the bacterial load was determined with LoD of 43 CFU/mL in food samples using the laser b. Copyright (2020), with permission from MDPI [94]. (**B**) Immunomagnetic separation with fluorescent-labeled sample and (**i**) video-processed using smartphone for detection of *Salmonella* with an LoD of 58 CFU/mL and (**ii**,**iii**) the efficiency of salmonella detection compared to other bacteria and bacterial-capturing mechanism with nanoparticle, respectively. Copyright (2019), with permission from Elsevier [95]. (**C**) Shin et al. presented a (**i**) CD-disk-type microfluidic system for lateral-flow assay, (**ii**) the assembly of lateral-flow assay, and (**iii**) multiplexed detection of *E. coli*, *Salmonella Typhimurium*, *Staphylococcus aureus*, and *Bacillus cereus* in contaminated lettuce samples. Copyright (2018), with permission from the American Chemical Society [96].

Microfluidic devices have facilitated lab-on-chip (LOC)-integrating micropores, mixers enhancing capture efficiency, micropillars, and microfilters as additional modules combining these analytical procedures onto the same chip. The miniaturization, portability, instant detection, automation, and high-throughput are key advantages offered by microfluidics that are widely applicative in sensitive detection of food pathogens and toxins [92]. Recently, many smartphone microfluidic platforms integrating immunomagnetic nanoparticles or urease enzyme or paper-based/impedance electrochemical measurements have been introduced, offering high-end food sensing with multiplexed and rapid detection of pathogens [93]. A study has reported QD fluorescent-probe-based readout integrated with manganese nanoflowers as QD nanocarriers for signal amplification to detect *Salmonella typhimurium*. The bacterial load was determined with a low detection limit of 43 CFU/mL in food samples such as chicken, depending on the fluorescent intensity of released QDs [94]. Another sensor introduced immunomagnetic separation with fluorescent-labeling and video-processing smartphone for detection of *Salmonella*. The immunomagnetic particles separated and concentrated *Salmonella* followed by labeling with immunofluorescent microspheres to form fluorescent bacteria. This fluorescent *Salmonella* was injected into a biochip integrated with a smartphone fluorescent microscopic system. A low detection limit of 58 CFU/mL *Salmonella* was obtained by online counting of fluorescent spots using a smartphone App. (as presented in Figure 3) [95]. Shin et al., recently proposed a lateral-flow assay for multiplexed detection of *E. coli*, *Salmonella typhimurium*, *Staphylococcus aureus*, and *Bacillus cereus* in contaminated lettuce samples (Figure 3c) [96].

Paper-based devices are facile and flexible analytical biosensors as they offer a wide range of advantages over microfluidic chips in being cost-effective, with easy fabrication, great biocompatibility and high capillary action [97]. Lateral-flow assays (LFAs) and microfluidic paper-based analytical devices (µPADs) are the most common type of paper-based devices. LFAs or dipsticks are known for their facile handling, and rapid and naked-eye-visible readout without any additional equipment. Their cost-effectiveness and versatility in assay formats and user-friendliness offer their wide applicability in point-of-care testing of food pathogens. LFAs simply comprise a sample pad where sample is added, a conjugate pad where the sample travels via capillary action activating the immobilized molecules, an absorbent pad, and a nitrocellulose membrane; all arranged on a plastic padding. The molecular components in the sample are separated as they travel across the membrane and produce a test line as positive-result output and a control line [98]. LFAs that are used for food-borne-pathogen detection incorporate monodispersed latex labels, gold colloid, and fluorescent/carbon tags for conjugate labeling. The colored particle, generally colloidal gold, binds to biomolecules (antigen/antibody/aptamer) immobilized onto test line that correlates with the amount of sample added [99,100]. Commercial LFA strips available in the market for bacterial sensing include *Listeria-*, *Salmonella-*, and *Escherichia coli* O157-Reveal test kits (Neogen^®^) Lansing, USA; *Listeria*, *Salmonella-* and *Escherichia coli*-VIP GOLD™ (BioControl Systems^®^) Bellevue, USA, and for *Listeria,* DuPont™ Lateral Flow System (DuPontQualicon) [4].

**Figure 4:**
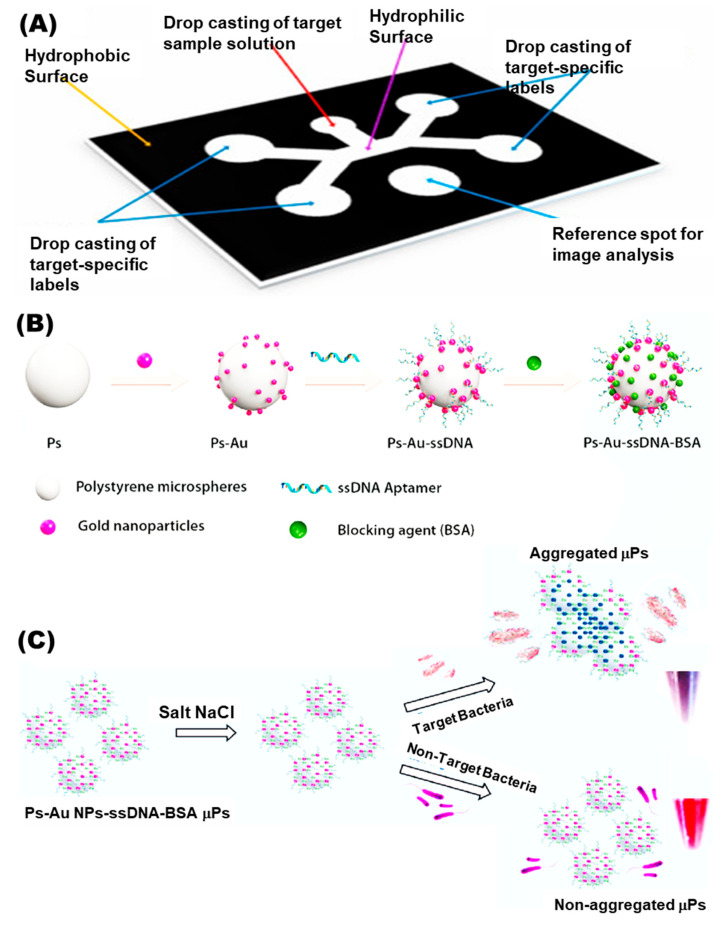
(**A**) Paper-based microfluidics assembly for multiplexed assay. (**B**) Nanoparticle surface modification and building with ssDNA and blocking BSA protein for detection of *E. coli* O157:H7 and *S. typhimurium.* (**C**) Sensor detecting whole-cell food pathogen with an LoD of 10^3^ and 10^4^ CFU/mL, respectively. Copyright (2022), with permission from Elsevier [101].

Paper-based μPADs generally utilize paper instead of chip microfluidics and are economical and efficient, removing the need for cleanroom facilities. Compared to silicon-based conventional biochips, paper-based chips are simple and highly porous, allowing physical absorption-generating devices that are easy to operate, modify and dispose of. These μPADs perform liquid transport, reactions, and even reagent storage on the hydrophilic porous paper that promotes transfer of liquids in the device. In this way, the designed flow-channels obviate the requirement for an external pump for running the assay [98]. The major component of paper μPADs is cellulose. Being biocompatible and flexible, it somehow absorbs the reagents dried onto it and arranged this in a cartridge integrated to a fluid delivery system viz. a droplet dispenser. Only the template has to be added to the kit and the start button is pressed, triggering the fluid delivery into μPAD. The fabrication of paper pads is categorized as patterning of hydrophobic barriers onto paper such as wax/laser/inkjet printing and shaping techniques, for instance, paper cutting/laser etching [98]. A recent work has developed an aptasensor integrating microfluidics paper-based multiplexed detection of *E. coli* O157:H7 and *S. typhimurium* (as presented in Figure 4). This novel sensor comprises single-input detection of more than single whole-cell food pathogen providing a quantitative signal readout as image analysis with a low detection limit of 10^3^ and 10^4^ CFU/mL, respectively [101].

Finally, we can say that the traditional approaches, such as PCR-based techniques and fluorescence detection on the surface are time-consuming and require specialized instrumentation. The microfluidic-based biosensor has shown its potential in research into rapid and sensitive detection with a very high limit of detection. Above, we discussed some examples of microfluidic biosensors for the detection of food contaminants. As add-ons to microfluidic systems and in integration with these methods, nanomaterials have become attractive in attaining selectivity. Nanomaterial provides a large surface area for binding of recognition molecules and enhances the signal for fluorescence. The use of nanomaterials in these biosensors makes them easy to use and feasible for point-of-care detection. In particular, the pros and cons of microfluidic-based biosensors include (i) high sensitivity in the analysis of small- and large-volume sample and (ii) high specificity and multiplexity to detect different analytes. In microfluidic systems, the challenges for food samples are is that some liquid samples are highly dense and cause blockage in the microfluidic device. Still, it is predicted that the future for microfluidic-based sensing of food samples is very promising.

## 5. Future Outlook

In this review, we have discussed the various research approaches, nanomaterials, and methodologies in fluorescent-based detection methods used for food safety. Traditionally, fluorescence-, and image-based biosensors are used to detect contamination in food and water. Food and water are very complex matrices, which not only include several diet elements (proteins, lipids, sugars, etc.), but also consist of parts such as additives. It is important to mention that fluorphores have the challenge of the aggregation-caused quenching (ACQ) effect, which restricts their function in sensing. The development of biosensors for food safety and their in-field application deal with issues pertaining to pre-treatment of complex samples such as the development of biosensors for food safety and their in-field application deal with issues pertaining to pre-treatment of complex food sample and maintain sensitivity. Moreover, a lower concentration of bacterial contamination in food samples is also challenging for target sensitivity and detection limits. Although microarrays are effective and accurate signal-producing technology, they require technical expertise and are expensive. Therefore, microfluidics or lab-on-chip devices hold great potential due to automation, miniaturization, and portability, and their ability to produce fast signal readout. However, certain limitations due to blockage of microfluidic channels or non-specific adsorption cause problems in complex sample analysis. In this context, signal-amplification methodologies, along with deep-learning strategies, can improve food-sensing fluorescent biosensing. Regardless of the performance of fluorescence- and image-based biosensors, they still have several challenges in real-world applications due to a high rate of false-negative or false-positive results and diet elements create autofluorescence and disrupt sensitivity and trigger false results. The nanomaterial-based fluorescent biosensors are able to address this problem. Although nano-biomaterials have benefits in operation, several parameters must be adjusted and need optimization. Extensive research, over several years, into sensing for food-safety purposes has shown that certain materials (e.g., graphene, metal nanoparticles) are usually preferred for fluorescence-sensing of food material. The advantage of using nanomaterials is the ability to achieve high signal intensity with selectivity. Nanomaterial-based biosensors have been successfully developed but suffer from constraints of stability, repeatability, and poor anti-interference ability. To overcome some major problems in fluorescence sensing, it is necessary to integrate and compare different methods to achieve optimum sensitivity. Chemometric, surface-enhanced Raman scattering (SERS), electrochemical sensing can also be used along with fluorescence for multiplexed sensing with high sensitivity. To date, fluorescent systems are in the experimental stage and practical functions of nanomaterial-based fluorescent biosensors in food matrices continue to remain under investigation. By implementing artificial intelligence and microfluidic systems for fluorescence biosensors we may achieve the goal of developing low-cost and real-time recognition of contaminants in food matrices. Recent research has shown the possibility of achieving sensitive and precise detection of food contaminants using the smartphone by enabling artificial intelligence for signal analysis without the requirement for sophisticated equipment. This development opens the door to a stand-alone, point-of-detection device for fluorescence-based detection, showing the possibility of detection of food contaminants outside the laboratory.

## Data Availability

Not applicable.
